# Intradural tumor and concomitant disc herniation of cervical spine

**DOI:** 10.4103/0019-5413.73663

**Published:** 2011

**Authors:** Mihir R Bapat, Prasanna Rathi, Uday Pawar, Kshitij Chaudhary

**Affiliations:** Spine Surgeon, Department of Bone and Joints, Kokilaben Dhirubhai Ambani Hospital and Medical Research Institute, Mumbai, India

**Keywords:** Intra-dural tumor, schwannoma, cervical disc herniation, disc simulating tumor

## Abstract

We report a rare patient of a simultaneous extradural and intradural compression of the cervical spinal cord due to co-existent intervertebral disc herniation and an intradural schwannoma at the same level. The intradural lesion was missed resulting in recurrence of myelopathy after a surprisingly complete functional recovery following anterior cervical discectomy. Retrospectively, it was noted that the initial cord swelling noticed was tumor being masked by the compression produced by the herniated disc. A contrast magnetic resonance imaging scan is important in differentiating intradural tumors of the spinal cord. A high index of suspicion is often successful in unmasking both the pathologies.

## INTRODUCTION

Cervical disc herniation is the commonest cause of cervical radiculo-myelopathy that requires surgical treatment. Decompression produces excellent results. Intradural tumors are rare. Their incidence is highest in the cervical spine. Due to their slow, indolent growth a high index of suspicion is required to diagnose these tumors. Co-existence of these pathologies is unreported and may confuse the physician. We report a case where an intradural tumor was missed due to the presence of a concomitant cervical disc protrusion.

## CASE REPORT

**Figure 1:**
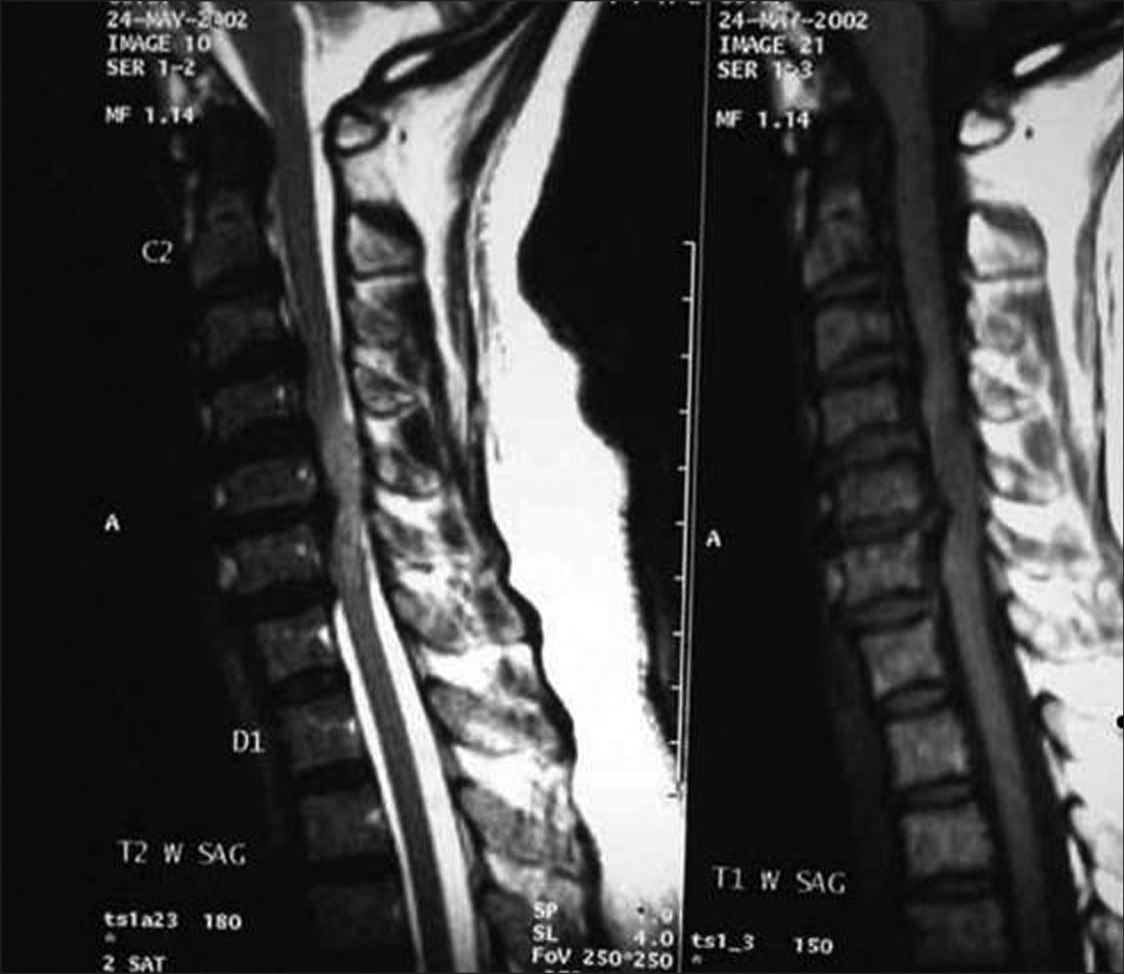
Preoperative sagittal TI T2 MRI images show disc herniation and the tumor

**Figure 2:**
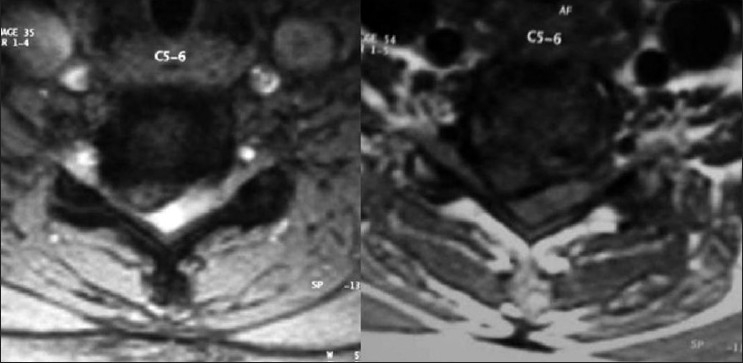
Preoperative axial T2W1 and T1W1 MRI images shows the disc herniation

A 55-year-old lady presented with clumsiness of fine motor functions of the hand and gait of two months duration. She was asymptomatic prior to this episode. On local examination there was no spasm, tenderness or deformity of cervical or dorsal spine. There was minimal spasticity and exaggerated deep tendon reflexes in all extremities. The plantars were extensor and the superficial abdominal reflexes were absent. The bladder and bowel functions were unaffected. The Japanese Orthopedic Association JOA score^1^ [Table 1] was 13/17; visual analogue score (VAS) for axial neck pain was 2/10. Medical co-morbidities were essential hypertension, hypothyroidism and chronic renal failure. The creatinine level was elevated at 2.4 mg/dl. The patient deteriorated rapidly in neurological function 24 hours after admission. The fine motor function of both hands was unaltered but the motor power in both lower extremities reduced to Grade 1 (Medical Research Council scale). The patient developed retention of urine and required catheterization. The sensory deficit increased in all extremities and also involved the trunk. The Japanese Orthopedic Association (JOA) score reduced to 4 /17. Preoperative radiographs showed redzuction in C5-6 disc height with slight reduction in the overall cervical (C2-C7) lordosis. The Pavlov’s ratio^2^ at C5-C6 was one (normal ratio is one and less than 0.82 is stenosis). There was no evidence of osteolysis or scalloping. Dynamic views ruled out instability. Magnetic resonance imaging (MRI) was performed after the sudden neurological deterioration. A C5-6 central disc herniation significantly compressed the cervical cord. A diffuse cord edema and swelling extended between C4 to C6 levels [Figures 1 and 2]. Due to chronic renal failure and elevated serum creatinine level, gadolinium contrast MRI was deferred.

**Table 1:** Japanese Orthopedic Association score

Criterion	Japanese Orthopedic Association score		
	On admission	After deterioration	Six weeks after ACDF
Motor function upper extremity	2 Fine motor function massively decreased	2 Fine motor function massively decreased	5 Normal function
Motor function lower extremity	5 Normal function	1 Unable to walk	5 Normal function
Sensory	2 Minimal sensory loss	0	2 Minimal sensory loss
Bladder function	4 Normal function	1 Urinary retention	3 Mild dysfunction
Total JOA score	13	4	15

JOA - Japanese Orthopedic Association; ACDF - Anterior cervical decompression and fusion

A diagnosis of anterior cord syndrome secondary to herniated cervical disc was inferred. An anterior C5-6 discectomy and uninstrumented fusion using iliac crest autograft was performed. The patient showed rapid recovery in the neurological status starting from the tenth postoperative day and regained independent ambulation by the third postoperative week. Bladder control was restored and the sensory deficit recovered. Patient was followed up regularly at six weeks, three months, six months, one year and two years. Follow-up at six weeks showed a JOA score of 15/17. Slight clumsiness of gait persisted till two years, which was perceived only on brisk walking (evident on detailed in depth questioning retrospectively). The patient also complained of urgency in micturition. The deep tendon reflexes remained exaggerated at two years. The lateral radiograph showed complete fusion of the graft.

**Figure 3:**
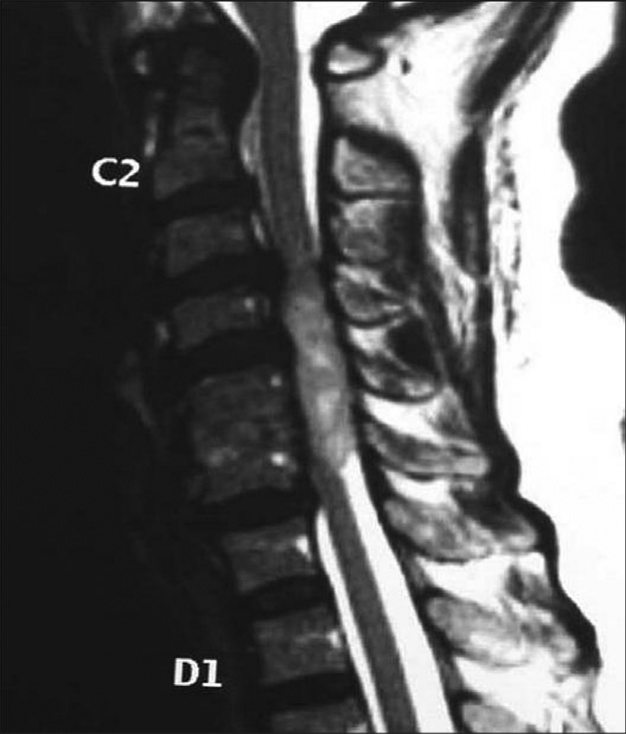
Sagittal T2 weighted image two years after ACDF done for recurrence of symptoms shows intradural extramedullary tumor

**Figure 4:**
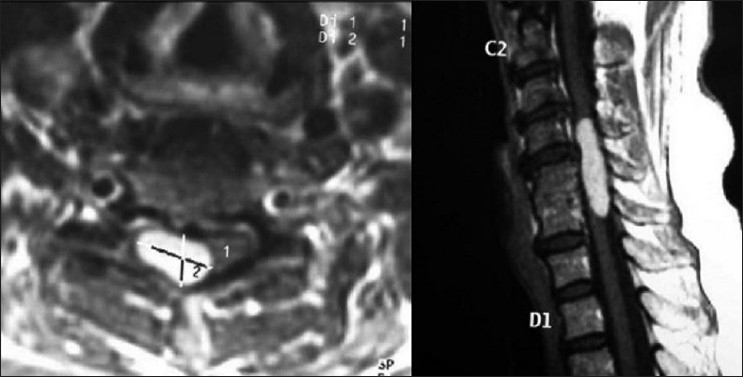
Postoperative postcontrast axial and saggital images shows intradural extramedullary tumor after two years of ACDF done for recurrence of symptoms

25 months postoperatively, the patient returned with increased symmetrical clumsiness of both hands and inability to walk unsupported on plain ground. The clumsiness of gait increased and was evident on routine daily activities. She complained of numbness in the upper extremities, trunk and the lower extremities. Bladder control remained status quo (slight urgency persisted). The JOA score was 7/17. A repeat MRI scan showed a right posterolateral 1.5 cm intradural tumor displacing the cord substance. The lesion was hyperintense on T1and T2 images. The tumor extended from C4-C6 [Figures 3 and 4] in the new contrast MRI that matched the extent of the cord edema noticed of the first MRI [Figures 1 and 2]. Patient’s creatinine was 1.8 mg/dl however contrast was used after consulting nephrologist as it was necessary. The liver functions were normal. The broad based central C5-6 disc protrusion obscured the delineation of the intradural tumor in the earlier MRI. The cervical cord showed myelomalacia. The tumor was separated en-mass from the cord substance through posterior laminectomy and durotomy approach. A water tight closure of the durotomy was performed.

On gross appearance the tumor felt soft and showed fine lobulation with a distinct pseudo-capsule. On the cut surface, the tumor appeared homogenous. The histopathology revealed a schwannoma with spindle shaped tumor cells and palisade pattern.

**Figure 5:**
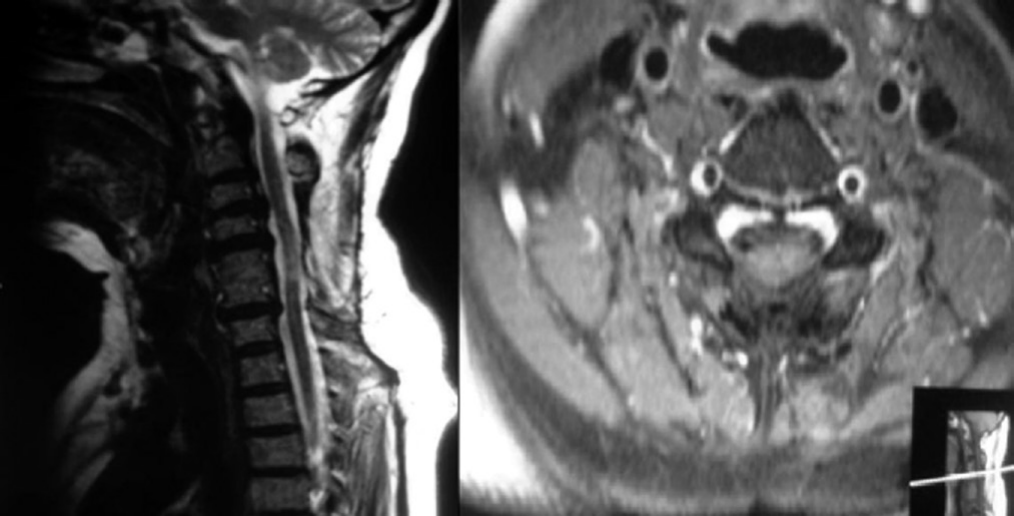
Two-year postoperative sagittal and axial MRI images after tumor excision

The neurological status improved rapidly and the patient regained the preoperative JOA score of 15/17 by the sixth postoperative week. Two years after the excision of mass, the patient remained slightly clumsy in hand function and gait but led a normal active life style [Figure 5].

## DISCUSSION

This case report highlights a rare co-existence of two significant pathologies contributing to cervical cord compression. It is uncommon for an acute cervical disc herniation to produce a profound acute neurological deterioration without causing axial or radicular pain. Following an anterior cervical discectomy, the compressive factor was reduced. The cord edema resolved allowing neurological recovery. Most intra-dural tumors follow a slow progressive, indolent course and there may be lag period of several months before symptoms become obvious.^3^–^5^ It can be hypothesized that a chronic intervertebral disc herniation pre-existed. The enlargement of the cervical cord by the tumor increased the pressure in a pre-existing stenotic canal akin to a compartment syndrome. This probably explained the absence of pain.

Schwannoma and meningioma are the commonest intradural extramedullary tumors.^3^–^5^,^8^ Enlargement of the cord substance is pathognomonic of an intradural enlarging lesion. A gadolinium contrast MRI helps to compartmentalize tumors into extradural, intradural and intramedullary varieties. Schwannoma differentiates from a meningioma by a peripheral rim enhancement on contrast images.^3^–^7^ The C5-6 disc herniation proved to be a red herring resulting in the intra-dural tumor being overlooked. The cord edema and swelling was noted in the first MRI but was overlooked as the incidence of two simultaneous pathologies is extremely rare and also a contrast MRI was not possible. A low index of suspicion along with the following reasons contributed to the missed diagnosis;

1. Acute neurological deterioration with involvement of the lower limbs more than the upper limbs (anterior cord syndrome)
2. Complete recovery after surgery allayed suspicion and deferred postoperative MRI
3. Definition of the tumor obscured by a broad based disc herniation in all images.
4. A gadolinium contrast MRI could not be obtained due to underlying renal disease.

Co-existence of a lumbar disc herniation and a proximal dorsal or cervical intradural tumor is reported.^11^ Simultaneous presence of upper and lower motor neuron signs in the extremities suggest the diagnosis. In case of lumbar intradural tumors, it is not uncommon for these lesions to simulate symptoms of a prolapsed intervertebral disc.^10^ In the present case both lesions compressed the cervical cord at the same level. As suggested in literature, recovery of myelopathy is satisfactory after decompression of the spinal cord in both these lesions. The astonishing aspect is the relative decompression effect produced by discectomy in this case that restored near normal function. The authors are unaware of a similar case report published in English literature. The incidence of anterior intradural tumors is the highest in the cervical spine. 3,9Since the two lesions were located differently (intradural and extradural), a separate approach was feasible.

## References

[1] Zasshi GSN (1994). Japanese Orthopedic Association scoring system for cervical myelopathy. Jap Ortho Asso.

[2] Pavlov H, Torg JS, Robie B, Jahre C (1987). Cervical spinal stenosis: determination with vertebral body ratio method. Radiology.

[3] Van Goethem JW, van den Hauwe L, Ozsarlak O, De Schepper AM, Parizel PM (2004). Spinal tumors. Eur J Radiol.

[4] Sevick RJ (1995). Cervical spine tumors. Neuroimaging Clin N Am.

[5] Takemoto K, Matsumura Y, Hashimoto H, Inoue Y (1998). Magnetic resonance imaging of Intraspinal tumors capability in histological differentiation and compartmentalization of extramedullary tumors. Neuroradiology.

[6] Pilavaki M, Chourmouzi D, Kiziridou A, Skordalaki A, Zarampoukas T, Drevelengas A (2004). Imaging of peripheral nerve sheath tumors with pathologic correlation: pictorial review. Eur J Radiol.

[7] Friedman DP, Tartaglino LM, Flanders AE (1992). Intradural schwannomas of the spine: MR findings with emphasis on contrast-enhancement characteristics. AJR Am J Roentgenol.

[8] McCormick PC, Post KD, Stein BM (1990). Intradural extramedullary tumors in adults. Neurosurg Clin N Am.

[9] Seppälä MT, Haltia MJ, Sankila RJ, Jääskeläinen JE, Heiskanen O (1995). Long-term outcome after removal of spinal schwannoma: a clinicopathological study of 187 cases. J Neurosurg.

[10] Wiesel SW, Ignatius P, Marvel JP, Rothman RH (1976). Intradural neurofibroma simulating lumbar-disc disease. Report of six cases. J Bone Joint Surg Am.

[11] Takeuchi A, Miyamoto K, Hosoe H, Shimizu K (2004). Thoracic paraplegia due to missed thoracic compressive lesions after lumbar spinal decompression surgery. Report of three cases. J Neurosurg.

